# Two-Eyed Seeing in action: Project extension for community health outcomes – Indigenous chronic pain & substance use

**DOI:** 10.1080/24740527.2025.2469213

**Published:** 2025-04-04

**Authors:** Andrew Koscielniak, Natalie Zur Nedden, Yaadwinder Shergill, Teresa Trudeau-Magiskan, Marinna Read, Alycia Benson, Lana Ray, Andrew Smith, Virginia McEwen, Paul Francis, Alex Falcigno, Tyler Drawson, Andrea Furlan, Christopher Mushquash, Patricia A. Poulin

**Affiliations:** a Indigenous Health, N’doo’owe Binesi, St. Joseph’s Care Group, Thunder Bay, Canada; b Department of Anesthesiology & Pain Medicine, Ottawa Hospital Research Institute, Ottawa, Canada; c Ka-Na-Chi-Hih (To Keep One Sacred), Thunder Bay, Canada; d Health Disciplines, Athabasca University, Athabasca, Canada; e Anishinaabe Kandaasowin Institute (AKI), Lakehead University, Thunder Bay, Canada; f Pain and Addiction Medicine, Centre for Addiction and Mental Health, Toronto, Canada; g Chronic Pain Management Program, St. Joseph’s Care Group, Thunder Bay, Canada; h Interventional Pain Service, Thunder Bay Regional Health Sciences Centre, Thunder Bay, Canada; i Family Medicine, Northern Ontario School of Medicine, Thunder Bay, Canada; j University of Toronto, Toronto, Canada; k KITE Institute, Toronto Rehab, University Health Network, Toronto, Canada; l Lakehead University, Thunder Bay, Canada; m Indigenous Health, Thunder Bay Regional Health Research Institute, Thunder Bay, Canada; n Dilico Anishinabek Family Care, Fort William First Nation, Canada; o The Ottawa Hospital Pain Clinic, Ottawa, Canada; p Faculty of Medicine, Department of Anesthesiology and Pain Medicine, The University of Ottawa, Ottawa, Canada

**Keywords:** Indigenous health, chronic pain, substance use, Two-Eyed Seeing, cultural safety, for project extension for community health care outcomes, Traditional Knowledge System, truth and reconciliation, Indigenous healing, health care inequities

## Abstract

**Background:**

Indigenous Peoples in Canada experience health disparities, including higher rates of chronic pain. Many report distrust of the health system due to factors such as racial discrimination. A lack of appreciation and respect for Indigenous knowledges further contributes to feelings of alienation. In 2022–2023, we offered the first Project Extension for Community Healthcare Outcomes (Project ECHO) Indigenous Chronic Pain and Substance Use Health (ICP&SU) to health care providers interested in improving chronic pain care with and for Indigenous Peoples in Canada. The program reflects a Two-Eyed Seeing approach weaving together Indigenous and Western approaches to chronic pain and substance use health care.

**Aims:**

We describe the development and implementation of Project ECHO ICP&SU.

**Methods:**

Following guidance from the project Elder, we use storytelling, centered around the metaphor of weaving, to discuss the conception and implementation of Project ECHO ICP&SU. We also describe our engagement in sharing circles and ceremonies to share stories, knowledges, and lessons learned.

**Results:**

With strong Anishinaabe leadership, the program was implemented as intended and reached 121 health care professionals. Lessons learned included an overt recognition of the influence of different structures and institutions on programs and for a culturally safer development and evaluation frameworks for future Project ECHOs to improve care with and for Indigenous Peoples.

**Conclusions:**

Project ECHO can be a vehicle to enact Truth and Reconciliation Calls to Action through weaving relationships and knowledges to create culturally safer institutions and practices to improve chronic pain, substance use health, and wellness, with and for Indigenous Peoples.

## Résumé

**Contexte :** Les peuples autochtones du Canada connaissent des disparités en matière de santé, notamment des taux plus élevés de douleur chronique. Un grand nombre d’entre eux font état d’une méfiance à l'égard du système de santé en raison de facteurs tels que la discrimination raciale.

Le manque d’appréciation et de respect envers les savoirs autochtones contribue également à ce sentiment d'aliénation. En 2022–2023, nous avons offert pour la première fois le programme ECHO (Extension for Community Healthcare Outcomes) sur la douleur chronique autochtone et l’usage de substances psychoactives (ICP&SU) aux prestataires de soins souhaitant améliorer les soins liès à la douleur chronique avec et pour les peuples autochtones du Canada.

Le programme repose sur l’approche de la « vision à deux yeux » qui tisse des liens entre les perspectives autochtones et occidentales en matiére de soins pour la douleur chronique et l’usage de substances psychoactives.

**Objectifs :** Nous décrivons la conception et la mise en œuvre du projet ECHO ICP&SU.

**Méthodes :** Suivant les conseils de l'Aîné du projet, nous utilisons la narration, centrée sur la métaphore du tissage, pour aborder la conception et de la mise en œuvre du projet ECHO ICP&SU. Nous décrivons également notre participation à des cercles de partage et des cérémonies de partage afin d’échanger des histoires, des connaissances et les leçons tirées de cette expérience.

**Résultats :** Grâce à un leadership Anishinaabe fort, le programme a été mis en œuvre comme prévu et a atteint 121 professionnels de la santé. Les leçons tirées incluent une reconnaissance manifeste de l'influence de différentes structures et institutions sur les programmes, ainsi que la nécessité d’èlaborer des cadres d'évaluation et de développement culturellement plus sûrs pour les futurs projets ECHO, afin d’améliorer soins avec et pour les peuples autochtones.

**Conclusions :** Le projet ECHO peut être un moyen de mettre en œuvre des appels à l'action de la Commission de vérité et réconciliation en tissant des relations et des savoirs pour créer des institutions et des pratiques culturellement plus sûres, visant à améliorer la douleur chronique, la santé liée à la consommation de substances psychoactives, ainsi que le bien-être, avec et pour les peuples autochtones.

## Background

### Locating ourselves

Boozhoo, hello, Biindigen, come in. Take a seat.

This article tells the story of the development and implementation of the first Project Extension for Community Health Outcomes—Indigenous Chronic Pain and Substance Use Program in Canada (Project ECHO ICP&SU). Project ECHO is a continuing professional development program that connects community-based health care providers (HCPs) to an interprofessional team of experts in a particular domain to provide case-based learning and didactic lectures via videoconference.^1^ Project ECHO has been implemented in many parts of Canada since 2014 but very few programs integrate First Nations, Inuit, and Métis perspectives and knowledges. Project ECHO ICP&SU sought to redress this, starting locally, by bringing Anishinaabe ways of knowing and doing with Western perspectives to advance CP understanding and care for Indigenous clients. The program was offered in 2022–2023 as part of a Health Canada Substance Use and Addictions Program–funded project. We wrote this article in response to many requests from health leaders interested in implementing similar initiatives in their institution.

Before we tell this story, we want to recognize the diversity that exists among Indigenous Peoples in Canada and the importance of a distinction-based approach in partnering initiatives to improve health outcomes for First Nations, Inuit, and Métis. We, the authors, recognize that our project took place at a particular time and space and that what we have learned needs to be understood within this context. This project brings together Anishinabek Peoples with strong community and cultural ties, Anishinabek Peoples currently learning about their culture and history and reestablishing relationships with community, White and racialized settlers, and people with adoption experiences working on retrieving their biological family, employed at different institutions. It also includes people with lived experience of chronic pain. Most activities took place at St. Joseph’s Care Group (SJCG) in Thunder Bay, on the territory of the Anishinabek Nation and home to Fort William First Nation, one of the signatories of the Robinson Superior Treaty of 1850. The project was directed by an Indigenous advisory board (IAB) consisting of Teresa Trudeau-Magiskan (Elder), Paul Francis (director, SJCG IHD), Marinna Read (clinical manager, SJCG IHD), Rosan Wesley (RSW, SJCG IHD), and Christopher Mushquash (C.Psych., Lakehead University, Dilico Anishinabek Family Care), all from or with solid ties to local First Nations communities (see the document Team Description in appendices). The project received full support from Ogichidaa Onaakonigewin, the Elders’ Council, which guides and oversees the work of N’doo’owe Binesi—Healing Thunderbird, the Indigenous health division at SJCG.

## Methods

### Choosing an appropriate methodology: Storytelling and weaving

It begins, as all things do, with stories^2^

Storytelling, as an Indigenous knowledge gathering and dissemination methodology, is relationally based in how it flows and functions within an Indigenous research paradigm. Storying promotes connection to traditional knowledges; healing spirit; creating, building, and maintaining relations; and creating new common understandings.^3^ When we engage in storying it speaks directly to “how you come to know begins with who you are, where you come from and the lens from which you see, interact, interpret, and make meaning out of the world. Keandossowin (translated as ‘coming to know’ from Anishinaabemowin) begins with how your experiences impact your learning journey.”^4,11^ When one is listening well, one must listen with all of their senses and be open to understanding that stories can be heard numerous times and meanings can shift and transform based on where you are at the time in your own growth and life circumstances.^3^ Further, storytelling is a process of reclaiming the narrative and meaning of the story, rather than the process of being defined by the story being shared.^5^

We utilize storytelling as a way to convey the wholistic and interrelated events, peoples, and processes that resulted in Project ECHO ICP&SU and to present our journey to establish and implement Project ECHO CIP&SU in a way that is relational, fostering localized critical reflection, and not a checklist approach.

When asked for guidance for the development of this article, Anishinaabe Elder Trudeau-Magiskan highlighted the connection between storytelling and weaving and shared: *The Indigenous customs of weaving to create tapestries with a story in them is a common practice of Indigenous communities of the Northwest Coast and in regions of North America by the Navaho and Pueblo. A common thread exists and connects family, community and nation within the regions where tapestries are made. The weavers create tapestries which have stories and they are identifiable by design, an image of a clan for example identifies the family and origin of the woven tapestry. The Navaho weavers share their stories, thoughts and prayers in woven blankets and rugs that were prized and sought after by other Indigenous Nations during the times of trading by Nations of North America.* For the reader, she stated: *As you read the story imagine a weaver is creating a tapestry and there are designs and colors that will produce a beautiful woven wall hanging, rug or blanket*. (Personal communication, Elder Trudeau-Magiskan, October 11, 2023). Later, Paul Francis, the then-director of Indigenous health—N’doo’owe Binesi, shared about the teachings of the dream catchers. Anishanaabek Peoples employ weaving in the construction of dream catchers, which are then hung in front of the window to catch bad dreams, allowing good dreams that contain messages and guidance to go through.

**Figure 1.**
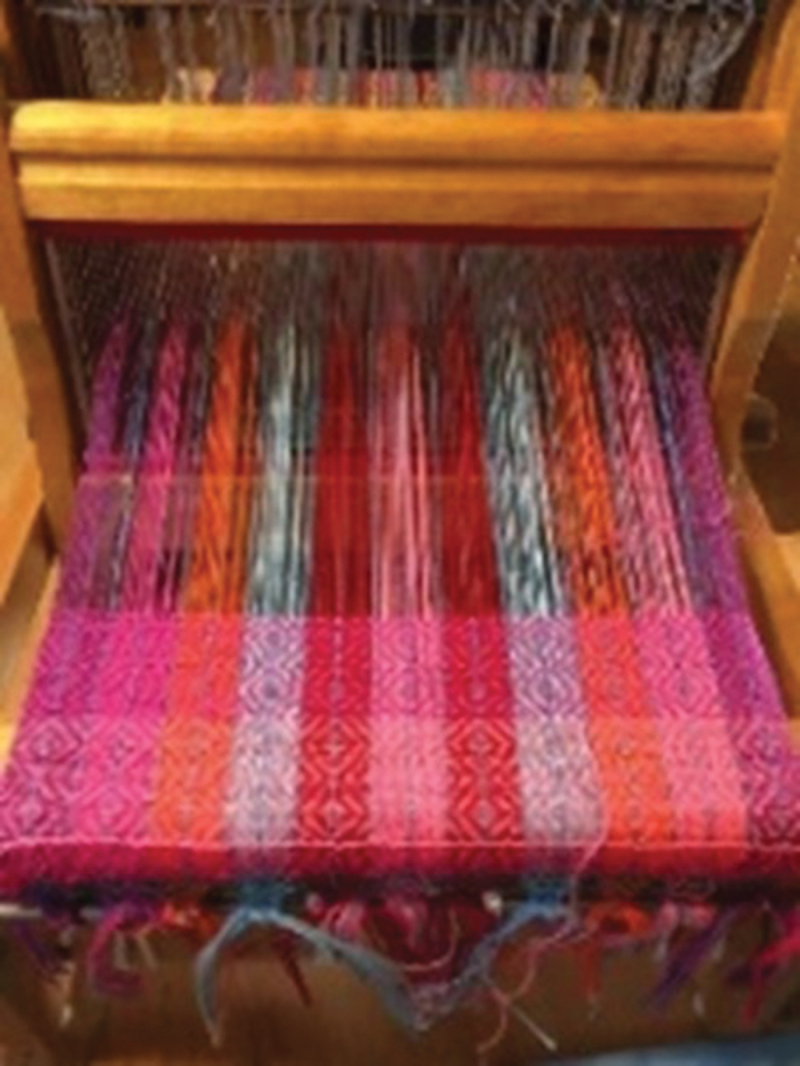
Modern loom table by Judy Kavanagh (weaver and person with lived experience of pain). Reproduced with permission.

Weaving is practiced in many First Nations, Inuit, and Métis communities across Canada, using different materials and techniques to weave different items such as blankets, mats, and baskets (Figure 1). Beyond its utilitarian purposes, which were emphasized by colonizers, weaving can serve as an avenue for socialization, cultural transmission, and spiritual connection and as a form of resistance to colonialism. Moreover, the act of weaving itself can be therapeutic, offering a meditative experience and a vehicle for healing.^6,7^

Project ECHO ICP&SU is a weaving project reflecting a Two-Eyed Seeing approach, which was coined by Mi’kmaw Elders Albert and Murdena Marshall, encouraging equal consideration of Indigenous and Western knowledges: “*Learning to see from one eye with the strengths of Indigenous ways of knowing and from the other eye with the strengths of Western ways of knowing and to use both of these eyes together*.”^8^ The intersecting stories of Indigenous peoples experiencing chronic pain, family members of those with chronic pain, health care providers bringing knowledges from different Western medicine disciplines and providing service to those with chronic pain, and Indigenous administrators who navigate Western health care systems came together to interweave their stories to design and implement Project ECHO ICP&SU. The program, like the dreamcatcher, sought to “catch” through story, evidence, critical reflection, and dialogue health service provider ignorance, bias, and assumptions so that training participants could be open to guidance that would allow them to grow the necessary knowledge and skills to provide and advocate for culturally safe chronic pain care for Indigenous clients. Like weaving, this project was therapeutic and challenging, creating space to engage in difficult conversations about the who, what, where, and how to implement culturally safety training for health service providers.

### Weaving: The basics

The loom is a device used by weavers to weave together threads to create a tapestry. The loom’s structure holds the warp threads under tension to facilitate the interweaving of the weft threads going over and under the warp threads, just as the framework of our traditions and worldviews holds our interconnected stories, values, principles, understandings, and experiences. The loom provides the structure for weaving, and its adaptability allows weavers to bring their creative visions to life. The range of possibilities is enabled by the resources, time, dedication, and skills of the weaver, serving as a powerful tool for crafting intricate and beautiful tapestries, each a visual story rich with cultural and personal meaning.

Our motivation for weaving is rooted in a commitment to redressing the health inequities experienced by Indigenous Peoples in Canada, starting locally and sharing with humility what we learned as we walked together. The loom, threads, weaving process, and resulting tapestry became metaphors for our project. The frame of the loom is made of wooden beams, representing the institutions and communities through which this project was possible. Binding cords secure the tapestry, symbolizing the agreements, connections, and relationships that brought our communities together for the project. To weave intricate tapestries, a multitude of colorful threads is essential; the threads represent the knowledges and experiences brought together by all participants.

Weaving requires the right level of tension: too tight and the threads risk tearing, too loose and the weave unravels. Similarly, our project required the careful management of tensions. The tension bar, used in some looms, symbolized our steadfast dedication to truth and reconciliation while also representing a degree of flexibility and adaptability. Unseen, the tension itself represents the discomfort we experienced as participants in the process, reflecting the underlying tensions fueled by history and current realities and our own personal journeys. As with any good story, we leave room for some mystery, discovery, and interpretation.

**Figure 2.**
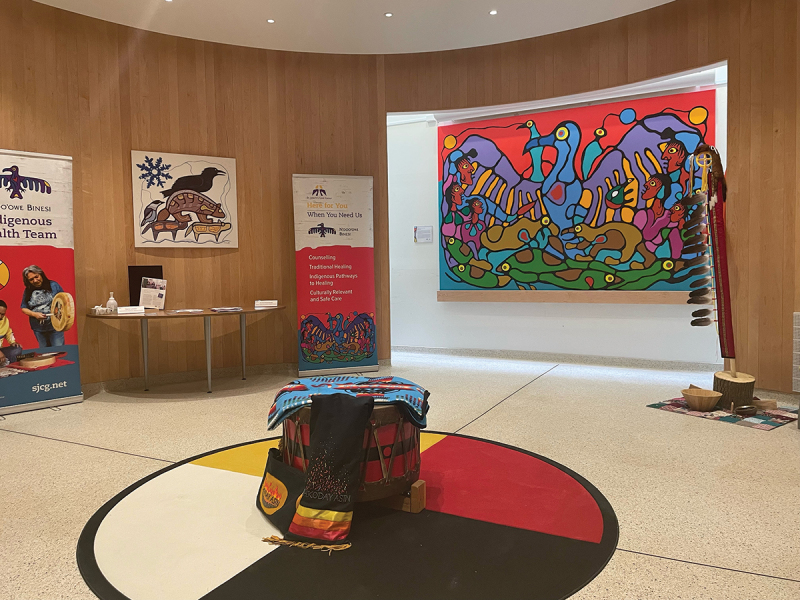
Spiritual gathering lodge. Andrew Koscielniak. Reproduced with permission.

With this in mind, we begin to tell the story of the first ICP&SU program in Canada and continue to unpack the weaving metaphor (Figure 2). This story unfolds in a fashion that is more linear than our lived experiences, much like a finished tapestry does not tell how much unweaving and reweaving might have occurred in the process of its creation or how the weaver carefully incorporated and transformed errors into features of the tapestry. As a team and individuals, we continue to learn. We hope that sharing our experiences will motivate others to weave their own tapestries to improve chronic pain care.

## Results—The story

### Why we weave: What is known about chronic pain among Indigenous peoples in Canada

Chronic pain is defined by the International Association for the Study of Pain as pain that persists or recurs for longer than 3 months.^9^ Chronic pain is ubiquitous; it is symptom of many conditions, but it is also now recognized in Western medicine as an illness in and of itself. Chronic pain affects one in five people in the settle nation now known as Canada.^10^ It can affect all dimensions of a person’s life, their families, and communities. The economic burden of chronic pain is substantial, with an estimated annual cost of $40 billion.^11^ Though Indigenous Peoples are strong and resilient, they are also disproportionately affected by chronic pain and various other health problems when compared to non-Indigenous Canadians.^12–15^ According to a Statistics Canada survey, Indigenous People represent the fastest growing population in Canada,^16^ and among those under the age of 65, they report the highest rates of CP.^17,18^ Furthermore, the probability of having at least one chronic condition such as diabetes or cardiovascular disease is also higher among Indigenous Persons. These chronic conditions can often cause or exacerbate a person’s chronic pain experiences.

Chronic pain has come into focus in Canada in 2019, when the then-Minister of Health Ginette Petitpas Taylor established the Canadian Pain Task Force under the Health Canada Cannabis and Controlled Substance Use Branch under which the Substance Use and Addictions Program exists. The task force was established to better understand and address the needs of people living with pain, in light of the rapid rise in opioid-related harms and deaths observed in Canada, the potential contribution of the rapid increase in opioid prescribing seen in the early 2000s on opioid-related harms observed, and changes in opioid prescribing guidelines. The increasing availability of toxic supply has also contributed to substantial harms. Although a minority of people living with pain who use opioids develop an opioid use disorder, the risk augments when exposure to violence, trauma, and unmet mental health needs coexist. Once more, although data are fragmented and limited,^19^ Indigenous Peoples in Canada have been disproportionally affected,^20^ leading several Indigenous communities across the country, such as Nishnawbe Aski Nation in Ontario,^21^ to declare states of emergency to address the opioid crisis and its impact on their communities.

The health disparities faced by Indigenous communities are deeply rooted in Canada’s colonial history and continue to be perpetuated by ongoing colonial policies and practices. Intergenerational trauma, systemic racism, food and water insecurity, displacement, loss of traditional knowledge and healing practices, and persistent economic inequities^22^ all contribute to the sustained marginalization of Indigenous Peoples. Colonialism is not a historical artifact but an ongoing reality that continues to shape the lived experiences of Indigenous communities both in Canada and across the globe.^22^ Indigenous Peoples often experience their pain as underreported, underassessed, and undertreated.^11,23^ Many Indigenous individuals are wary of the health care system and fearful that disclosing pain symptoms will lead them to being labeled as “drug seekers” or “malingerers.”^11,18^ This mistrust may be fueled by many factors, including intergenerational trauma,^24^ racial discrimination, and poor treatment by HCPs,^25^ which have sometimes resulted in harm and avoidable deaths.^26^ The socialization processes embedded in intergenerational trauma, along with a historical and ongoing context of discrimination and mistreatment, influence how Indigenous Peoples express pain and interact with the health care system.^27,28^ This is compounded by the fact that Westernized medicine privileges specific epistemologies and ways of healing that may not align with Indigenous Peoples’ traditional knowledge systems and healing practices. This epistemological racism is alienating for some Indigenous Persons. The marginalization of Indigenous healing practices and the lack of cultural safety in the health care system further complicate the experience of pain management for Indigenous Peoples.^8,29^ Cultural safety, in this context, refers to an environment where Indigenous Peoples feel respected and safe, free from discrimination, and where their cultural identities, values, and practices are recognized and integrated into care.^30^

### Indigenous and Western knowledge systems in chronic pain care

The Two-Eyed Seeing approach has been applied in many fields, including chronic pain.^31^ Though its roots are Mi’kmaw, the framework has been adopted by some Anishinaabe scholars^32^ and health leaders. It offers an avenue to advance a more collaborative approach that recognizes the strengths of Indigenous and Western knowledges.

From Indigenous perspectives, pain is conceptualized as “a part of life” and holistic, including interrelated physical, emotional, and spiritual dimensions.^33,34^ Whereas physical and emotional pain are more commonly understood, spiritual pain can refer to pain experienced because of settler colonialism and genocide, which can manifest physically and emotionally.^34^ Further, emotional pain resulting from racism, settler colonialism, dispossession, and dislocation can significantly impact physical health, including physical expressions of pain, experienced by Indigenous Peoples.^25^ Because spiritual and relational components of pain that are rooted in oppressive systems of Indigenous erasure and intergenerational trauma ripple throughout communities, contributing to poorer health outcomes,^35^ pain can also be understood as a collective experience.

Within the Western medicine dominant paradigm, pain is generally viewed as an individual problem that needs to be eradicated. Although the emotional and mental dimensions of pain are increasingly being recognized, pain often remains conceptualized primarily as a physical experience and is assessed using tools that do not recognize the complex interactions of the emotional, physical, mental, and spiritual dimensions of pain.^36^ The emphasis on the physical dimension of pain has led to significant advances in understanding and treating chronically painful conditions (e.g., various forms of arthritis, sickle cell disease, various types of migraines), and identifying the contributions of various pain generators has galvanized innovation in pharmacological and interventional pain medicine approaches.^37–39^ However, many Indigenous Peoples are not benefiting from these advances due to the aforementioned issues.^40,41^ For example, one study focusing on Mi’kmaq children found a propensity toward hiding pain and being stoic, leading to delays in the diagnoses and treatment of various health conditions.^40^ A recently qualitative study found that discriminatory attitudes and systemic barriers continue to affect Indigenous Peoples’ access to and experience of primary care.^41^ Without access to primary care, access to specialized chronic pain treatment is almost impossible. Further, specialized care is condensed in areas of high population density, and travel is not always possible given time away from family and other duties.^41^

Western models of understanding pain such as the biopsychosocial model,^42^ fear avoidance model,^43^ and neuromatrix theory of pain^44^ recognize the complexity of peoples’ experiences of pain beyond biological factors, including attention to the role of chronic stress, cognitive appraisals of pain, and social determinants of health, among others.^11^ More recently, the role of trauma in the etiology and experiences of chronic pain is being increasingly recognized.^11^ As such, interdisciplinary or interprofessional approaches to pain management are now being recognized as the gold standard.^45^ However, access to such care remains difficult.^46^ Further, these models and approaches are anchored within Western frameworks that remain focused on individuals^47^ and do not reflect Indigenous ways of knowing and healing. As a result, Indigenous Peoples’ needs are often not met, and they report that they do not feel safe accessing services within the Westernized health care system.^48–50^

There is extensive knowledge within Indigenous communities on traditional healing and medicines utilized to treat pain that are rarely recognized by Western HCPs.^51^ This includes the use of plant medicine to treat the different dimensions of pain (e.g., it is common knowledge in Indigenous communities that willow bark is used for physical pain and sage is used for emotional and spiritual pain) and ceremonial healing, which are often used in conjunction with one another.^51^ Further, from Indigenous ways of knowing, pain can have multiple meanings; it can be an important messenger, a visitor, a teacher, an offering, or a test, and turning away from the message of the pain can lead to suffering. Emergent from Indigenous concepts of pain, Indigenous practices for pain management take a holistic approach to identify and treat pain, recognizing the connections between mind, body, spirit, and emotions.^33^

Recognizing the aforementioned challenges and in alignment with the Truth and Reconciliation Commission of Canada—Calls to Action (TRC #22),^22^ the Canadian Pain Task Force calls for the acknowledgment and integration of traditional Indigenous healing practices, knowledge, and medicine in pain care for Indigenous Peoples.^11^ Both the TRC and the Canadian Pain Task Force also call for the use of trauma- and violence-informed approaches in Indigenous-led health partnerships to advance care for Indigenous Peoples,^22^ and both emphasize the need for improved training and education for HCPs to ensure cultural competency (TRC #23).

Our recognition of the health disparities facing Indigenous Peoples in accessing chronic pain care, our appreciation of the epistemological discord observed, and the value of a Two-Eyed Seeing approach, along with the pull of the TRC calls to action, created the necessary tension for our team to start weaving and working on the development of the first ICP&SU.

### The first frame: Project ECHO within Canadian institutions and arriving in Thunder Bay

Project ECHO is both an institution and a movement that aims to improve access to health care, with a particular attention to people living in remote, rural, or underserviced areas.^52^ It is a continuing professional development program that connects community-based HCPs to an interprofessional team of experts in a particular domain to provide case-based learning and didactic lectures via videoconference.^1^ Project ECHO was developed in New Mexico^52^ in 2003 to improve the management of hepatitis C in rural New Mexico and because of its success has since been reproduced and adapted across a variety of diseases and specialties around the world.^53,54^ Project ECHO provides a proven structure to “move knowledge, not people,” using an “all teach, all learn” basis where humility and openness are key. A systematic review of 39 studies across 17 illnesses (including chronic pain) revealed that Project ECHO increased knowledge and self-reported competence among HCPs. Health system outcomes included improved health care access and cost-effectiveness.^55,56^

In 2014, A. Furlan of the University Health Network, in collaboration with R. Dubin of Queen’s University, received funding from the Ontario Ministry of Health and Long-Term Care to implement the first Project ECHO Chronic Pain & Opioid Stewardship in Canada. The ECHO Chronic Pain & Opioid Stewardship Hub is dually located at the University Health Network and Queen’s University. The program has significantly impacted the management of chronic pain and opioid use across Ontario by increasing the capacity of HCPs to deliver evidence-based care.^57^ This has been achieved through enhanced knowledge and self-reported competence among HCPs, as demonstrated in various studies that highlight the program’s effectiveness in improving patient outcomes and changing provider behaviors.^58–60^

In 2017, recognizing the success of the University Health Network ECHO Program and the need for a second hub of HCPs experienced in remote, rural, and underserviced areas, the Ministry of Health funded Project ECHO Chronic Pain and Opioid Stewardship St. Joseph’s Care Group (Project ECHOSJCG), a partnership between St. Joseph Care Group in Thunder Bay and The Ottawa Hospital in Ottawa. Through ongoing program evaluation activities, the team identified a significant gap: there were no curricula focusing on Indigenous chronic pain, and there was limited participation from Indigenous HCPs and settler HCPs working in Indigenous health settings. Aware of this gap, we developed the first Indigenous Chronic Pain ECHO Series.

### Weaving the first ICP&SU tapestry

The first Project ECHO series that focused on Indigenous chronic pain at SJCG was developed in a silo. There was no relationship between SJCG Project ECHO and the Indigenous Health team. The leadership team of SJCG Project ECHO at the time (all of whom have now moved to other positions, with the exception of Poulin) turned to the people and resources they knew in the field, none of whom were local at the time. The Indigenous CP ECHO consisted of a three-session series that was offered in February 2020. These sessions emphasized the following key topics: traditional healing practices, fostering meaningful allyship, and understanding opioid use disorder and chronic pain in Indigenous communities. In addition to the hub members, 16 health care professionals attended the sessions. Participants reported improvements in their abilities to engage in conversations about integrating traditional healing approaches in their practice, forming allyship with Indigenous clients experiencing chronic pain, and discussing opioid use disorder with Indigenous clients. Though this initiative reflected the commitment of SJCG Project ECHO leadership to enhancing culturally appropriate care and addressing specific needs within Indigenous communities, there was no clear path forward to ensuring an ongoing sustainable series addressing this. The tapestry was not well secured, the structure too loose. Now we see that the proper frame for this work was not in place, but unbeknownst to SJCG Project ECHO leadership, construction was underway.

### Building a more solid frame allowing the weaving of a second ICP&SU tapestry

From 2018 to 2021, under the leadership of the Francis, director of Indigenous relations, SJCG launched “Wiidosem Dabasendizowin: Walking with Humility A Plan to Develop Relationships and Practices with Indigenous Peoples.”^61^ Following the Medicine Wheel and starting in the Eastern Direction, representing infancy, this plan set the strategic direction to improve Indigenous health services at SJCG, aligned with the Truth and Reconciliation Calls to Action #22 and 23. This included commitments to improve cultural safety through training, strengthen Indigenous voice in the institution through an Elders’ Advisory committee—Ogichidaa Onaakonigewin, and increased access to traditional healing for Indigenous clients. This also included concrete actions to build relationships with community (e.g., Mushkiki Aboriginal Health Access Center). This work created a critical part of the frame needed to anchor a second Project ECHO ICP&SU tapestry, and it is ongoing.

In 2021, Health Canada provided funding to expand the Project ECHO initiative across the country through a financial contribution awarded to Project Lead Furlan at University Health Network, under the Substance Use and Addictions Program. Based on the lessons learned from the first Project ECHO ICP&SU, and in alignment with the Canadian Pain Task Force’s recommendations on Indigenous chronic pain, coapplicant Poulin proposed allocating a portion of this funding to develop and pilot a specialized Indigenous Chronic Pain and Substance Use ECHO program. For sustainability, this program would ideally be connected with an existing ECHO program focused on chronic pain and substance use, leveraging its established infrastructure. Additionally, it was crucial for the program to be housed within an institution with a clear and demonstrated commitment to truth and reconciliation. Project ECHO at SJCG was identified as a fitting partner to provide a solid frame for this work given its prior success in piloting a brief Indigenous Chronic Pain ECHO series and commitment and dedication to advancing Indigenous Health. Poulin approached SJCG Project ECHO supervisor Koscielniak, who then partnered with the SJCG N’doo’owe Binesi, Indigenous Health Department Director Francis to advance the project.

One of the many important roles of the Indigenous Health Department is to provide leadership and direction for Indigenous initiatives, support partnership building, support the development of Indigenous programs, and ensure cultural competency with SJCG with the approach of cultural humility as a journey. Cultural humility, in this context, refers to a lifelong process of self-reflection and learning in which HCPs actively challenge their own biases, recognize power imbalances, and engage in respectful partnerships with Indigenous communities.^62^ Together, Koscielniak and Francis secured the necessary institutional approvals for SJCG to host the first formal Project ECHO Indigenous Chronic Pain & Substance Use in Canada. Through formalizing these partnerships, the frame of the loom was now solid enough; we could begin tying the warp threads representing the various knowledges and principles needed for an ICP&SU ECHO series, bringing together Indigenous and Western ways of knowing and doing, reflecting a braided approach to chronic pain care.

Reflecting back on the process, we acknowledge that the connection between all of the people involved in their respective roles was a common goal of improving Indigenous health outcomes. However, in hindsight, what was missing were the relationships between project ECHO leadership and Indigenous health leaders as well as community engagement with Indigenous organizations, which would have facilitated a partnered approach in line with the “Nothing about Us without Us” precept.^63^ The Project ECHO ICP&SU arrived shortly after the N’doo’owe Binesi started its second phase of Wiidosem Dabasendizowin, Walking with Humility, moving from the Eastern (infancy) to the Southern (adolescence), with more relationship building planned. The Health Canada funding opportunity provided the impetus and resources required to have the necessary conversations between Project ECHO ICP&SU and N’doo’owe Binesi that allowed partnership, mutual learning, and transformation to occur.

Though on the surface it appeared that White settlers (A.F., P.P., A.K.) were proposing a project to Indigenous health leaders without having consulted with them first, the reality was far more nuanced, again due to colonialism and anti-Indigenous racism. At the time, Project ECHO at SJCG and N’doo’owe Binesi were operating in parallel, without connection. When P.P. raised that the path forward would require strong Indigenous partnership and leadership, Project ECHO SJCG Supervisor Koscielniak, a registered kinesiologist with a master of arts in leadership, had 7 years of frontline experience providing services within the SJCG chronic pain management program and had been a supervisor of Project ECHO SJCG for 2 years, took what was a very courageous step for him and disclosed his Indigenous status as a member of the Nippissing First Nations. This was a pivotal moment that galvanized the relationship between Project ECHO and the Indigenous Health Department at SJCG. Through a series of individual and team meetings, the project received full leadership support from SJCG’s Addiction & Mental Health, Community Mental Health Department, and N’doo’owe Binesi (Indigenous Health Department). Furthermore, the project received support and approval from Ogichidaa Onaakonigewin, the Elders Council at SJCG.

The Project ECHO ICP&SU is the result of more than 10 months of partnership building and knowledge exchange activities between Project ECHO leadership and the Indigenous Health Department at SJCG, as well as with Indigenous clinicians, scholars, administrators, and Elders located in different institutions and communities, as well as further learning for Project ECHO team members.

### Dyeing the threads: Training and knowledge exchange for project ECHO staff

Through conversations and knowledge exchanges, gaps were identified among Project ECHO staff members. It became clear that further training would be necessary for the Project ECHO ICP&SU to deliver a program integrating the strengths of Indigenous and Western knowledges and reflecting the historical and current realities underlying chronic pain and substance use among Indigenous Peoples. Staff and leaders of Project ECHO at SJCG leadership completed training on Indigenous Health and cultural safety including Repairing the Sacred Circle: An Indigenous Cultural Awareness and Education Primer offered by SJCG IHD. This training focused on concepts such as colonization, care and racism, and common stereotypes and how these contribute to health inequalities for Indigenous People. It is also an introduction to Indigenous Knowledges and cultural protocols and practices, such as the use of the Sharing Circle and traditional medicines. ECHO staff also completed the San’yas Indigenous Cultural Safety Ontario Core Health training. The San’yas curriculum is designed to help participants strengthen their knowledge, awareness, and skills for working with and providing service to Indigenous Peoples and communities; work more safely and effectively with Indigenous Peoples; and begin considering their role in correcting, rebuilding, and transforming systems to uproot Indigenous-specific racism (San’yas Antiracism Indigenous Cultural Safety Education, 2024).^64^

Much like preparing the threads for a vibrant tapestry, acquiring knowledge requires careful preparation. Indigenous ways of knowing emphasize the importance of selecting the right fibers, akin to identifying relevant knowledge sources. These materials must be cleansed and prepared, mirroring the need for unlearning to occur and approaching new information with humility and openness. The dyeing process takes time. Fibers are immersed in a bath that is sometimes heated slowly to a simmer. As the dyed fibers are hung to dry, we allow the new insights to settle and become part of our understanding, adding rich hues to the overall tapestry.

### Weaving the outside edges: Forming additional partnerships, establishing ICP&SU governance, hub, and curriculum

As the fibers dried, we worked on new partnerships to inform the development of the ICP&SU curriculum and ensure its accreditation for continuing medical education. This included the important step of convening the ICP&SU IAB. All of this work was led by Koscielniak, a member of the Nipissing First Nation, in his role as Project ECHO supervisor and member of the SJCG Indigenous Health Education Committee. Between April and November 2021, Koscielniak reached out and met via videoconference or phone with representatives from the following 12 organizations:

- Northern Ontario School of Medicine
- N’doo’owe Binesi, Indigenous Health Department, St. Joesph Care Group
- Thunder Bay Regional Health Sciences Center
- Dilico Anishinabek Family Care
- First Nations, Inuit and Metis Wellness ECHO, Center for Addiction and Mental Health
- Center for Research Aging and Health (CERAH), Lakehead University
- McGill Health Sciences Center Project ECHO
- North West Regional Rehabilitation Care Program, St. Joseph Care Group.
- Sioux Lookout Meno Ya Win Health Center
- Northern Indigenous Health Benefits (NIHB) & Jordan’s Principle
- Lakehead University
- Center for Addiction & Mental Health

These meetings were focused on further building relationships, learning more about the needs and wants of Indigenous Peoples and about how the First Nations, Inuit, and Métis Wellness ECHO ran. Through these conversations, Koscielniak also recruited members for the IAB.

The IAB focused on generating a diversity of views for the Project ECHO ICP&SU pilot team to consider in its process of developing, implementing, and evaluating this pilot program. The IAB consists of Teresa Trudeau-Magiskan (Elder), Paul Francis (director, SJCG IHD), Marinna Read (clinical manager, SJCG IHD), Rosan Wesley (RSW, SJCG IHD), and Dr. Christopher Mushquash (C.Psych., Dilico Anishinabek Family Care). They are responsible for providing advice on strategic direction, stakeholder engagement, needs assessment, and curriculum development, promotion, evaluation, communication, and sustainability planning. The board met regularly between June and October 2021 and was involved in all aspects of the pilot, including working with the ECHO Scientific Planning Committee to develop the curriculum as well as ongoing reflections about the session and impact of the ECHO, providing opportunities for further weaving Indigenous Knowledges into the project. The IAB also provided input into a community engagement survey that was circulated to over 30 hCPs working with or in Indigenous communities (previous Project ECHO Chronic Pain and Opioid Stewardship participants, SJCG regional rehabilitation staff, etc.) in July 2021. Seven people responded, and the information they provided was reviewed and incorporated with the other sources identified above to inform the development of the series. The IAB was also key in constituting the Project ECHO ICP&SU hub.

### Envisioning the tapestry: Core teachings for settler health care providers

The IAB identified six core components of the curriculum to be delivered over the ten sessions constituting the first series: (1) understanding trauma through an Indigenous lens, (2) history of the opioid crisis and its impact on Indigenous Peoples, (3) clinical monitoring and opioid tapering, (4) psychological pain and its physical manifestations, (5) assessment of chronic pain and substance use through an Indigenous lens, and (6) traditional approaches in chronic pain and substance use management. The last session consisted of a sharing circle.

The second series was modified by the IAB due to changes in the availability of the hub team; the sessions focused on the history of the opioid crisis and its impact on Indigenous Peoples were replaced by sessions on systemic racism, cultural safety, and pain and navigating the NIHB system. The session on clinical monitoring and opioid tapering was replaced by a session focusing on pharmacological pain management more broadly.

To encourage as much participation/attendance as possible, the series was certified by the Continuing Education and Professional Development Office at the Northern Ontario School of Medicine for up to 15 Mainpro+ credit(s) for the College of Family Physicians of Canada and was an Accredited Group Learning Activity (Section 1) as defined by the Maintenance of Certification Program of the Royal College of Physicians and Surgeons of Canada for a maximum of 15 h. The program learning objectives aimed to enable participants to (1) perform a culturally sensitive assessment and collaboratively develop an evidence-informed chronic pain care plan, recognizing that social determinants of health and previous conditions (e.g., historical and intergenerational trauma, stigma, racism, fear) may contribute to the chronic pain experience; (2) explain the history and impact of opioids on Indigenous Peoples and how to safely prescribe and manage analgesics for chronic pain, identifying both traditional and conventional resources available in the community to develop an effective local approach to prescribing analgesics for chronic pain that minimizes substance misuse, enhances treatment, and promotes safety; and (3) describe the bio/psycho/social/spiritual complexities of chronic pain and how to guide Indigenous People to both traditional and conventional chronic pain management options.

The Project ECHO ICP&SU hub is constituted of an interdisciplinary team bringing together Traditional Knowledge Keepers, clinicians trained in various health care disciplines, and clinicians who carry Traditional Knowledge while also being regulated health care professionals. Teresa Trudeau-Magiskan, Indigenous cultural health associate at SJCG, was the hub Elder, recognized by Ogichidaa Onaakonigewin (SJCG Elder’s Council). The session facilitator was Karen St. Jacques, a settler and physiotherapist with the SJCG Chronic Pain Management Program with client outreach experience across Northwestern Ontario. The ECHO Hub also consisted of hub physicians Virginia McEwen and Andrew Smith and hub pharmacist Jana McNulty (RPh); hub psychologist Chris Mushquash for the first series and Patricia Poulin for the second series; and hub social worker Rosan Wesley for the first series and Valerie Shawinimash and Marinna Read for the second series. Tasha Toulouse, from noninsured health benefits, also joined the hub for the second series. The hub held a sharing circle at the end of each session to discuss shared learnings and guide the next session. For example, after the first case discussion, which used a more traditional biopsychosocial approach, Project ECHO Elder Teresa Trudeau-Magiskan brought the Medicine Wheel into focus to guide future case discussions. The following case discussions were all approached with the Medicine Wheel as a framework.

### Weaving the second ICP & SU tapestry

Following the above-described curriculum, we delivered the ten sessions of our first pilot series between January 1 and March 31, 2022. A total of 80 people (including 26% nurses, 13% social workers, 10% physicians) from seven provinces registered into the program. There was an average of 32 participants from different disciplines such as medicine, nursing, physiotherapy, and chiropractic per session. A change in Project ECHO and N’doo’owe Binesi leadership as well as the demands placed on hub members put some tension on the frame, but we were still able to deliver a second pilot series between January and March 2023 with some adjustments to the curriculum and a slightly different hub team. We had 41 participants (15% nurses, 10% physicians, 5% social workers) across six provinces.

All case discussions were focused on Indigenous clients. To ensure that the cases were viewed through a Two-Eyed Seeing lens, integrating Indigenous and allopathic approaches, the hub Elder reminded attendees to keep in mind the four quadrants of the Medicine Wheel (spiritual, physical, mental, and emotional aspects of health and treatment) while discussing differential diagnoses, additional investigations to perform (e.g., blood work, imaging), and suggestions for treatment (e.g., pharmacotherapy, physical therapy, psychotherapy, use of Traditional Medicines, and participation in Ceremonies). Hub physicians, physical therapists, and psychologists, along with program participants, brought forward their respective perspectives to address the patient/case presenter goals. We evaluated the series through surveys and interviews, discussed the results with our IAB, and shared some findings with members of the community. These will be the subject of a subsequent manuscript.

### Sharing and taking in the beauty, embracing the mistakes and the learning

After the two series were delivered, the IAB met to discuss the series, review evaluation data, and discuss implications and next steps. As storytelling intends, the delivery of the core curriculum and case discussions intends to touch the hearts and minds of attendees, hub members, and staff and, as we highlighted at the beginning, weaving requires a careful management of tensions on the frame and the threads.

In the IAB meeting, we discussed opportunities to improve the program through providing participants with materials that could help them better prepare for the session, including information about residential schools, the Sixties Scoop, and other relevant historical facts as part of the registration process.

Discussion with the IAB also included attention to ensuring that future series convey how the Medicine Wheel is both congruent with and distinct from the biopsychosocial approach. This could be done through an emphasis on the fact that the teachings offered during the sessions were introductory and through highlighting points of congruence and distinctions.

The IAB also discussed how we could foster cohesion and a strong sense of community among hub members dispersed across Ontario and who, in some cases, never worked together. The discussion also highlighted the importance of coming together as a group to further reflect on learnings and continue the work, and this led to an event we called “Sharing Day.”

**Figure 3.**
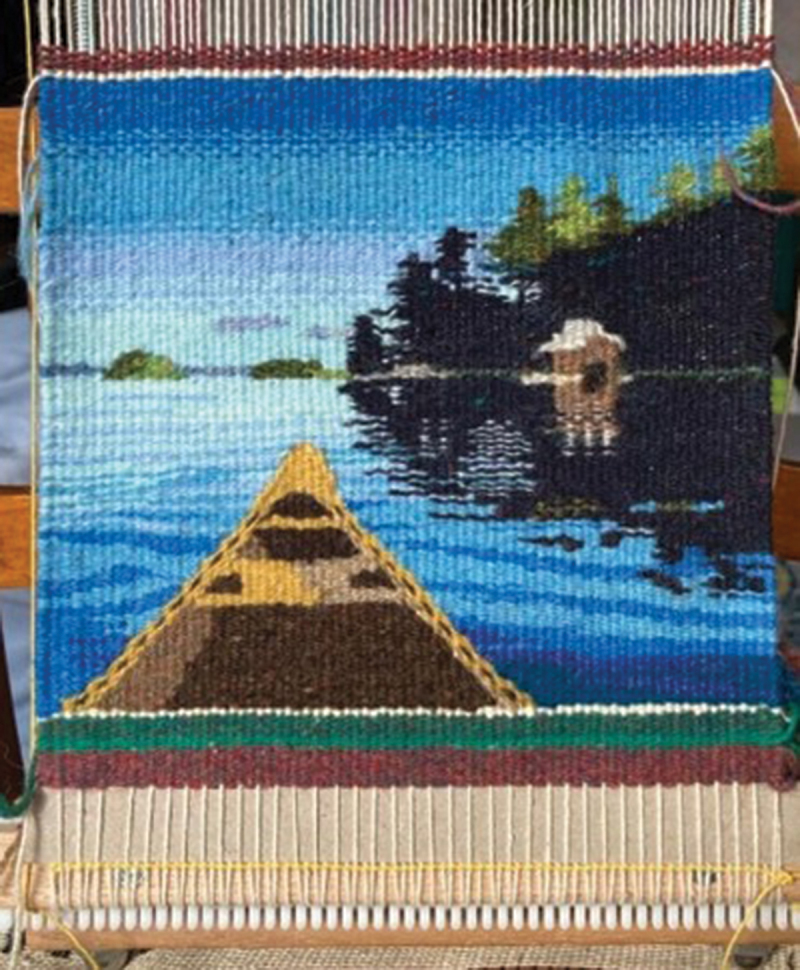
Canoe and cottage tapestry. Judy Kavanagh. Reproduced with permission.

The Sharing Day brought together Project ECHO leaders (locally and nationally), Project ECHO ICP&SU hub members, members of the IAB, and members of N’doo’owe Binesi (Indigenous Health, Wellness, and Partners Division, SJCG). We came together first to review our foundation through participating in *Repairing the Sacred Circle: An Indigenous Cultural Awareness & Education Primer*, led by Elder Ron Linklater, where we heard stories of trauma and resilience in relation to colonization, discrimination, and challenging experiences in health care. We reflected on our respective locations, histories, and intentions, as well as on the meaning of truth and reconciliation: “*Reconciliation is coming together to look at the past together to move forward together in a good way*. In our 900 language this conveys a beautiful message of reconciliation—and to remember this as our country and people start their healing journey” (Elder Ron Linklater, Sharing Circle, November 30, 2023) (Figure 3).

The sharing day included Ceremonies (Smudging, Prayers, Drumming), Talking Circles, didactic presentations, and discussions, as well as teachings from an Elder and Cultural Knowledge Keepers reflecting a braided approach to sharing knowledge and experiences in a good way. Though the day had a structured agenda, the group was committed to being adaptable and flexible and welcomed impromptu teachings that will not be written about out of respect for the essence and process. We reflected on learnings, strengths and weaknesses, and different worldviews for pain care. We also reflected on the question of *What is a good outcome* for future Project ECHO programs with regards to improving health outcomes with and for Indigenous Peoples.

## Discussion

### Reflecting, learning, and making the best of weaving mistakes

This project was part of a larger initiative meant to improve chronic pain and substance use health care in Canada through continuing professional development for health care professionals and was funded through a Health Canada Substance Use and Addictions program grant. The program was delivered as intended and reached over 100 hCPs, mostly situated in Ontario. However, we noted many limitations and lessons learned, some of which have been highlighted throughout the article and will not be repeated here.

The first limitation we highlight is the fact that a deficit-based approach to improving health care outcomes with and for Indigenous peoples has been highly criticized given that it can further solidify deficit-based narratives and fuel stigmatization.^65^ Further, the connection between chronic pain and substance abuse is problematic. These are two stigmatized problems.^66^ This is in part due to the fact that the project was funded by the Substance Use and Addictions program within the Cannabis & Controlled Substance Branch at Health Canada. Though a holistic approach is preferable to improve the health and well-being of people living with chronic pain and people who abuse substances, programs focusing on this intersection run the risk of contributing to this confluence. Nevertheless, HCPs rate chronic pain, mental health, and substance abuse as being the most challenging conditions to address in their practice, and Project ECHO is one of the vehicles through which we can improve knowledge and practices, and it can also be a vehicle through which a deficit-based approach is challenged through elevating Indigenous voices and ways of knowing,

Another challenge we encountered was the high demands placed on the Indigenous health leaders, impacting availability of hub members for the second series. As a result, the session curriculum and the hub changed between the first and second series. Further, new hub members from different cities across Ontario needed to be recruited on very short notice; team members did not all know each other and met for the first time a few minutes before the first session, and there was no backup in case of absences. Given the central role of relationality in many Indigenous cultures and given the sensitivity of the curriculum, adequate time needs to be dedicated to ensuring strong connections within the hub who will be responsible, much like the dream catcher, to “catch” bias and assumptions and let through the transformative teachings that will allow participants to improve their understanding of Indigenous Peoples’ experiences of health care, pain, healing, and wellness and ability to provide and advocate for culturally safer chronic pain care for Indigenous clients.

Our evaluation of the program will be the subject of another paper. However, we highlight that the evaluation was driven mostly by the funding agency evaluation matrix. Though very supportive of the pilot project and not in opposition to the evaluation required through the granting agency agreement and for continuing education credit, an important issue that has been identified by Indigenous researchers and members of our IAB is the appropriateness of the evaluation framework for this program. Kawakami (2007)^67^ highlighted that evaluation activities must take place in the context of a specific place, time, community, and history and promote and practice Indigenous worldviews. Though our approach to the development of the Project ECHO ICP&SU is largely aligned with the Public Health Agency of Canada’s Aboriginal Ways Tried and True Framework,^68^ our approach to its evaluation was not, for many understandable reasons. The most important one is that the necessary relationships for this to occur were not in place at the time of the initial development of the proposal for funding, but the resources provided allowed for these relationships to be formed and sufficiently nurtured for the Project ECHO ICP&SU delivery. We committed to redressing this, partly through the integration of the Sharing Day into our process. Further, our team, supported by funding from the Canadian Institutes of Health Research, has been working on identifying the core components of a culturally safe ICP&SU development and evaluation framework.

In addition, Churchill et al.^69^ asserted that client/patient outcomes must be measured over time to accurately assess the extent to which cultural safety training programs improve how Indigenous Peoples are treated by HCPs. Work is ongoing at SJCG to design and develop a more robust and integrated evaluation strategy for Indigenous health initiatives; however, at the time of the study, such an evaluation was not feasible.

## Conclusion

We have shared the story of the development of the first ICP&SU program in Canada following a Two-Eyed Seeing approach. We worked with the metaphor of weaving as suggested by Elder Teresa Trudeau-Magiskan. The process of writing the story helped us deepen our understanding of the “complexities and inter-relatedness of each important strand of the process.”^70^ It also challenged us to embody some of the Medicine Wheel teachings as we came together to write about the process and outcomes of the Project ECHO ICP&SU with intention and respect, and we reflected on how we could do things differently in the future. The tapestry we weaved continues to make impressions that we are documenting, and this is motivating us to continue to work toward strengthening Project ECHO’s capacity to be a vehicle for truth and reconciliation in Canada. Elder Brenda Mason led the Sending Off ceremony for this article as we offered good thoughts and prayers, as well as offerings of tobacco to the land. We thank you for taking the time to sit with us. Miigwetch.

## Supplementary Material

2025 01 27 Supplementary Material Team Member.docx
