# Supplementary material for: Two-Eyed Seeing in action: Project extension for community health outcomes – Indigenous chronic pain & substance use
Source: Can J Pain. 2025 Apr 4;8(2):2469213. doi: 10.1080/24740527.2025.2469213 (PMC11980454; doi:10.1080/24740527.2025.2469213)
Supplement: 2025 01 27 Supplementary Material Team Member.docx [file UCJP_A_2469213_SM3954.docx]

**Team Description**

**Andrew Koscielniak**

**Boozhoo, Shkoday Mukwa indizhinikaaz,**

Heron dodem,

Nbissing indoonjii,

My Anishinaabe name is Fire Bear, my English name is Andrew Koscielniak, I use he/him pronouns.

I am a member of the Heron clan and a member of Nipissing First Nation. I am of Ojibway and Algonquin descent, my People have lived in the area of Lake Nipissing since time immemorial. My mother’s mother was Anishinaabe and my mother’s father was an Irish settler. My father’s parents were both Polish settlers. I recognize the unearned privilege I have received from my settler background and my presentation as white. I am aware that I walk in two worlds and one-path experiences less resistance.

I grew up in Robinson Huron Treaty territory, In Sudbury, ON. Spent my summers on Nipissing First Nation territory. From the time I was born until 14 y/o I walked the path of a proud Anishinabee youth, I celebrated all my cultural backgrounds, I had no fear or shame of this.

At 14 years old that changed, my dad was transferred for work to Robin Superior Treaty territory or Thunder Bay. This was the first-time I ever experienced racism.Within a week of moving to Thunder Bay my mother told my siblings and I to not tell anyone we are Indigenous. We didn’t. Because I don’t look Indigenous, people were openly racist around me all of the time. I didn’t say anything, I didn’t know how. I did what I was told to do, I learned quickly to hide who I am, to be fearful and shameful. I continued to do this for over 20yrs

When I was hired with SJCG in 2013, in the Chronic Pain Management Program, I continued to hide who I am. I didn’t feel safe to tell the truth, I was fearful of the response from my colleagues. I continued to experience racism and discrimination in the workplace. Back then, racism and discrimination wasn’t talked about at work, Indigenous culture wasn’t present, spaces weren’t safe.

Fast forward to 2021 when I started to work with SJCG organizational Indigenous leaders, Paul Francis Jr., Reena Larabee & the N’doo’owe Binesi team on a the Project ECHO Indigenous Chronic Pain and Substance Use pilot project that I began to feel safer to talk about my Indigenous culture.

I engaged in cultural safety training at SJCG where we openly talked about racism and discrimination in healthcare. I joined the Indigenous Health Education Committee (IHEC), SJCG, where I learned more about my culture, participated in Indigenous cultural education across the organization and began to feel safer to speak the truth about who I am as a person to other people.

My journey hit a personal breaking point in Sept 2022, at the SJCG National Day for Truth & Reconciliation Sacred Fire closing ceremony, where after listening to the Elders share their stories, I spoke to one of the Elders about my story of hiding, fear, and shame. I broke down in tears, I just couldn’t hide anymore, I knew I needed to be honest about who I am. After that, I looked for more opportunities to learn about my culture. This opportunity came in late fall 2022, when a position with N’doo’owe Binesi was posted. In Nov ’22, I joined the Indigenous Health team – this is where I started to really lean into my healing journey. I joined an amazing team of strong Indigenous People who were doing amazing things to make change for other Indigenous People (clients and staff), so they don’t need to hide, feel afraid, or shameful of who they are, like I was. As a member of this team, I began to feel safer and safer in all spaces across the organization to be who I am, to speak the truth. At the Sacred Fire in 2023, as co-chair of IHEC I spoke in front of >50 people at the opening ceremony. I felt entirely safe to speak the truth about who I am as an Anishinabee person.

I attribute my feeling of cultural safety to the no-stop hard work and dedication N’doo’owe Binesi has put into Indigenous Cultural Safety & Humility, to our allies who stand beside us and support us on this journey, and SJCG as an organization for their dedication to Truth & Reconciliation.

Since then, I no longer hide who I am at work, I am not affair to speak the truth about my culture, I no longer feel shame to tell the people of SJCG who I am.

- I am Shkoday Mukwa, Fire Bear

- I am a member of the Heron clan, and Nipissing First Nation

- And I am proud to be Anishinabee

**Miigwech**

**Andrew Koscielniak**

**Paul Francis Jr., M.S.W., R.S.W.**

My Anishinaabe name is ShkodeManidooBinesi, which translates to *Fire Spirit Thunderbird*. I am Anishinaabe, and my Doodem (clan) is Mukwa, the Bear. I come from Manidoo Msning (Manitoulin Island) and am a registered member of Wiikwemkoong Unceded Territory. Born and raised in Thunder Bay, Ontario.

My late mother, Lana Marie Francis (Desmarais), was of French and mixed European ancestry. My father is Paul Francis Sr. (Eagle Bear), and my grandmother, Rita Eshkawkogan (Ozawanemkii)-ba, was from Wiikwemkoong. My Grandfather, Kenneth Francis (Nanabush)-ba, was raised in Whitefish River First Nation (Birch Island).

I am the proud father of Royal, Harlow, Ailee, Siinese, and my adopted son Tristian, with my wife, Kyla. I hold the cultural responsibilities of Pipe Carrier, Sun Dancer, and work to support the revitalization of our ceremonies and culture. As part of my spiritual duty, I assist in caring for Piimmii-Gabow the Grandmother Eagle Staff at N’doo’owe Binesi, and in return she spiritually watches over and cares for us.

I serve as the Vice President of N’doo’owe Binesi, Indigenous Health, Wellness, and Partnerships at St. Joseph’s Care Group (SJCG), where I am dedicated to advancing Indigenous health equity and advocating for our Treaty Right to healthcare and Traditional Healing Services.

**Sat Sri Akal, I am Yaadwinder Shergill,**

I am a first-generation Canadian with Indian roots, and I use she/her pronouns. My parents are proud Sikhs, and I was raised in a home where the principles of seva (selfless service), compassion, and humility shaped who I am today.

I currently live in Milton, Ontario, on the traditional and treaty lands of the Mississaugas of the Credit First Nation, part of the Treaty 19 territory. I acknowledge the stolen lands on which I live and work and recognize the deep, enduring connection that Indigenous Peoples have to this land. As an ally, I am committed to listening, learning, and actively engaging in efforts that support justice, reciprocity, and healing for Indigenous communities.

While I am not Indigenous, I stand in solidarity with the Indigenous Peoples of this land. I am mindful of the history of colonization, the impacts of displacement, and the ongoing fight for sovereignty and rights. In my role as an ally, I strive to amplify Indigenous voices, acknowledge historical injustices, and work toward a more equitable and respectful future. My Sikh faith teaches me that true justice comes from seeing all people as one, and I carry this principle forward in my support for Indigenous communities.

Growing up as a Sikh Canadian, I experienced the complexities of navigating multiple identities. This experience has fueled my empathy and understanding of the challenges faced by Indigenous Peoples in Canada. I believe that allyship is about standing alongside those whose rights and culture have been marginalized, and I am dedicated to amplifying Indigenous perspectives and advocating for systemic change that prioritizes cultural safety and equity.

As a chiropractor and researcher focused on chronic pain, I work to ensure that my practice is inclusive, culturally sensitive, and patient-centered. I am committed to learning from and supporting Indigenous knowledge systems and working collaboratively with Indigenous communities to ensure that health and wellness are accessible to all.

It is my belief that true progress comes when we embrace humility, listen to those who have been silenced, and stand united for justice. I am proud to walk this path with the understanding that allyship requires continuous action, reflection, and a deep respect for the ongoing struggles and resilience of Indigenous Peoples across this land.

**Virginia McEwen**

My name is Virginia McEwen, nee Portmann, and I am a white settler. My parents are immigrants from Switzerland and made their home on a farm in Alberta, neighbouring the traditional territory of the Four Nations of the Maskwacis. I experienced the privilege afforded by my white skin, and although one of my closest friends growing up was Indigenous, I was oblivious to effects of colonialism that disadvantaged him and simultaneously allowed me to thrive.

As a young nurse, I chose to seek employment as a community health nurse in Northwestern Ontario Indigenous communities out of a sense of adventure. I felt the nursing curriculum at the time was progressive and prepared me for being a cultural ally, when in reality I was unaware of the ongoing embedded systemic biases. I spent 2 and a half years working in the North Caribou Lake First Nation and deeply loved this community. This community is the reason I sought a career in medicine, in order to have the autonomy to provide better care. The Band Council of this First Nation wrote one of my letters of recommendation to the Northern Ontario School of Medicine (NOSM). I wish to publicly acknowledge my gratitude for this, and that the spirit in which they supported my bid for a medical career is likely a strong reason why I was successful in achieving entry. Yet I now reflect this is an example of another white person advancing themselves on the backs of good Indigenous people. I also have had the fortune to serve in the Northwestern Ontario communities of Sandy Lake First Nation and Kashechewan First Nation for short periods of time. I went on to work for 2 years in the community of Pond Inlet on Baffin Island in Nunavut, with short periods in Qikiqtarjuaq and Kugluktuk. Working with the Inuit allowed me to appreciate the vast richness of culture within the land we call Canada.

In medical training at NOSM, the curriculum has a social mandate to improve care for the North, with a special focus on Indigenous populations. Although I had lived in Indigenous communities for several years, I still did not know half of the history or the far-reaching impacts of colonialism. I am now convinced that I still know very little, and continue to learn.

I now live in Thunder Bay, originally known as Anemki Wequedong, and now also known as the Treaty Territory of the Fort William First Nation. I am grateful to live, work and play on this land. I am an uninvited guest that has much to give back to this community and its people, and humbly thank the Anishnawbe, this land and its creatures, and the Creator for their gifts.

**Alycia Benson**

*Boozhoo, Aanii*

*Alycia Benson nindiznijaaz*

*Nigig Nindoodem*

*Ma’iigan Nindoodem*

*Biigtigong N’indoon-jii*

*Thunder Bay Nindaa*

I am an *Anishinaabe'Kwe scholar,  Biigtigong  N’Indoon-Jii* (the place where the river erodes) of the *Nigig nindoodem* & *Ma'iigan nindoodem* (otter clan & wolf clan), which is located off the shoreline of *Chi-Gamig* (Lake Superior). On my maternal grandmother's side, our relations are to *Biigtigong Nishnaabeg*; on my maternal Grandfather's side, we have ancestral relations to the James Bay Cree communities in northern Ontario. I also have relations on my paternal grandmother side in Europe. I am of both *Anishinaabe'Kwe* and settler. I live within a duality and diverse representation of identity.

Miigwetch

Alycia Benson, PhD(c), MA, HBa

**Marianna Read**

Marinna Read is a registered Fort William First Nation community member and resides in Thunder Bay. She holds a bachelor’s degree in criminology from the University of Manitoba and an honours bachelor of social work and master of social work degrees from Lakehead University. She is also an elected council member for the Ontario College of Social Work and Social Service Workers.

She has spent over 20 years practising in youth and adult mental health, Indigenous child welfare, program and community development and is currently the Clinical Manager at Ka-Na-Chi-Hih Healing Lodge. She has committed her professional career to assisting those on their wellness journey in a respectful and kind way ensuring culturally safe care through an Indigenous lens.

Her maternal family has Ojibway ancestry and are registered members of Fort William First Nation and later in life discovered origins in the Algonquins of the Greater Golden Lake area. Her Paternal family moved from Italy to Canada three generations ago.

Her personal life includes plenty of family time and enjoying the great outdoors in beautiful northwestern Ontario.

**Lana Ray nindizhnikaaz. Waaskone Giizhigook niintigo, Oshowkinoozhe n’dodem, Opwaaganasiniing nindoonjibaa.**

Hello, my name is Lana Ray. My spirit name is “the light that shines”. I am a member of the muskellunge clan. I am from the place of the pipestone (aka Red Rock Indian Band).

I am an Associate Professor and Canada Research Chair in Resurgent Methodologies for Indigenous Health at Athabasca University and the co-Director of the Anishinaabe Kendaasiwin Institute (AKI). Previously, I was an Associate Professor in the Department of Indigenous Learning and Indigenous Research Chair in Decolonial Futures at Lakehead University. Before that I worked in the public and not-for profit sectors, including as the Director of Policy and Research at a provincial Indigenous organization.

Also update on the advisory committee. The ED of KKETS, a fellow researcher and I met with Dennis Wiindigo for a day about two weeks ago and are hoping to have a clearer vision of things regarding an advisory committee ask soon.

Happy Winter Solstice!
